# The Impact of COVID-19 on Parental Perception of Oral Health-Related Quality of Life of Children: A Comparison of a Sample from Saudi Arabia and Kuwait

**DOI:** 10.1155/2023/9983979

**Published:** 2023-07-18

**Authors:** Wasmiya A. AlHayyan, Abdulrahman D. AlSaffan, Maram I. Alenezi, Bashaer K. Almutairi, Lena F. Alammari, Sharat Chandra Pani

**Affiliations:** ^1^Ministry of Health, Government of Kuwait, Kuwait, Saudi Arabia; ^2^Department of Preventive Dental Sciences, College of Dentistry, Riyadh Elm University, Riyadh, Saudi Arabia; ^3^College of Dentistry, Riyadh Elm University, Riyadh, Saudi Arabia; ^4^Schulich School of Medicine and Dentistry, University of Western Ontario, London, Canada

## Abstract

**Materials and Methods:**

OHRQoL was measured using a a validated Arabic version of the Child Oral Health Impact Profile (COHIP). Parents of children aged between 5 and 9 years were administered questionnaire during the COVID-19 pandemic. The responses were compared across the different domains of the questionnaire between the two countries using the Mann–Whitney *U* test. Differences were also tested between the parents of males and females separately in each city. The correlation of the COHIP scores with the age of the child was done using the Spearman's rho.

**Results:**

No significant differences in overall COHIP scores were found between the parents in Riyadh and Kuwait City (*p* > 0.05). There were significant gender differences observed across domains in Kuwait (*p* = 0.030) but not in Riyadh (*p* = 0.295). There was also a significant negative correlation between the different COHIP domains in Kuwait but not Riyadh.

**Conclusion:**

There is a greater gender difference and age correlation of OHRQoL among the population studied in Kuwait City when compared to those in Riyadh.

## 1. Introduction

Oral diseases, especially dental caries, have negative impacts on children and their families' functional, social, and psychological well-being [1–3]. The negative impacts of oral diseases include pain, discomfort and difficulty in chewing, loss of appetite, weight loss as well as difficulty in sleep, low self-esteem, and unsatisfied school performance [2, 4]. These impacts on the quality of life have been collectively termed as the oral health-related quality of life (OHRQoL) and has been used to effectively study how oral health can affect the well-being of an individual [5]. In children, the parental perception of their OHRQoL has been used as a proxy for their own self-reported oral health outcomes.

The oral health of children in the Gulf Region has shown to be poor. In Kuwait, the prevalence of oral diseases, including dental caries and periodontal disease, is high, affecting 61% of preschool children [6]. This is also true in Saudi Arabia where the prevalence of dental caries has been placed as high as 83% in preschool children [2, 7, 8]. This creates a challenge for oral health professionals to reduce the dental caries burden and provide dental care to the population. It also creates a burden on public expenditure on oral healthcare in both countries. Previous research has shown that the high burden of oral diseases creates unique OHRQoL challenges in the Gulf Region, with data from Saudi Arabia documenting some of these challenges [2, 6–8].

The COVID-19 pandemic has been recognized as having created disruptions in the overall health and well-being of children across the world [9–11]. The early years of the pandemic (2020–2021) saw the suspension of all nonessential dental procedures as the world sought to understand the impact of droplets and aerosol on the spread of the disease [11]. This led to suspension or modification of dental treatment in dental clinics across the globe [12, 13]. The reduction in dental care has been recorded among dental clinics in both Saudi Arabia and Kuwait [14, 15]. There is, however, little known of how these reductions have impacted the oral health, and more specifically the OHRQoL, of children in these two countries.

The child oral health impact scale—Child Oral Health Impact Profile (COHIP) is a validated method that allows the measurement of the perception of child's OHRQoL via a structure questionnaire composed of 34 questions in five major domains [8]. The Arabic version of this scale has been previously validated and used successfully to measure parent-reported OHRQoL of children in the region [16, 17]. Given this background, the aim of this study was to compare the parentally reported OHRQoL of children aged 5–9 years old in two cites, Kuwait City, Kuwait and Riyadh, Saudi Arabia, during the total restriction of dental care at the peak of the COVID-19 pandemic.

## 2. Materials and Methods

### 2.1. Patient Selection

The participants were randomly collected from patients list of the pediatric dentistry clinic in Riyadh Elm University Hospital, Riyadh city, Saudi Arabia, and pediatric dentistry department at Al Jahra Specialized Dental Center (ASDC), Kuwait aged between 5 and 9 years. All patients lived within the city limits of the respective centers. The patients had presented to the clinic before March 2020 and had returned to the clinic after pandemic restrictions had been lifted.

### 2.2. Evaluation of OHRQoL

Parents were administered a previously validated Arabic version of COHIP after obtaining informed consent. Data were collected between February and March 2022. The data were then collated into a spreadsheet (MS Excel, Microsoft Corp., Palo Alto, CA, USA) for analysis. The COHIP domains, namely, self-image, school environment, socioeconomic well-being, functional well-being, and oral symptoms were scored and used to assess the components of OHRQoL. Furthermore, the overall COHIP scores were used as a global measure of OHRQoL.

### 2.3. Statistical Analysis

The *t*-test was used to compare age differences between groups and genders, while the Mann–Whitney *U* test was used to compare differences in the OHRQoL between groups. The relationship between age and OHRQoL was measured using the Spearman's correlation coefficient. All statistics were performed using SPSS version 23 data processing software (IBM SPSS, IBM Corp., Armonk, NY, USA).

## 3. Results

The parents of a total of 756 children (466 males, 290 females) aged between 5 and 9 years (mean age 6.8 ± 1.3 years) were administered the COHIP questionnaire. A total of 511 parents in Riyadh and 245 parents in Kuwait City responded to the questionnaire. When the demographic profile of the population was compared, it was observed that there was no significant difference in age between male and female children in Kuwait City but not in Riyadh (Table 1).

**Figure 1:**
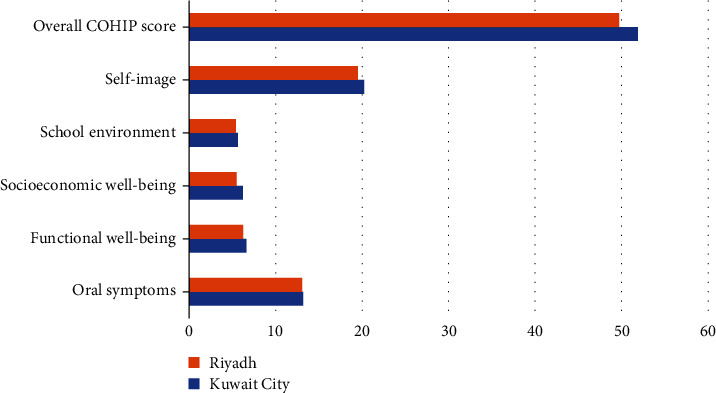
Comparison of the different domains of COHIP between Riyadh and Kuwait City.

When the COHIP scores were compared across domains between the two cities, it was observed that the parents of children in Kuwait City consistently reported higher mean scores than parents in Riyadh across domains (Figure 1). This difference was only statistically significant in the self-image domain (Table 2).

When gender differences in OHRQoL were compared, it was observed that there were no significant differences in overall scores between males and females in Riyadh but not in Kuwait City (Table 3). There were higher scores for females over males in Kuwait City in the self-image domain (0.019) but not in the other domains (Table 3). In Riyadh, the OHRQoL scores for males were higher than that for females, although the differences were not statistically significant (Table 3). When the impact of age on the different domains of OHRQoL was compared, it was observed that OHRQoL scores decreased with age in both Riyadh and Kuwait City. However, the correlations were significant only in Kuwait City not in Riyadh (Table 4).

## 4. Discussion

The role of parent-reported OHRQoL as a proxy for their children is an area that has received much attention in literature [18–20]. Previous work in the region has seen the COHIP scale used to successfully compare OHRQoL between children in Kuwait and Saudi Arabia [20]. Given that the COVID-19 pandemic has been shown to cause disruptions to dental care in both countries, this study aimed to compare the impact that such a disruption could have had on the parental reported OHRQoL in the two countries. The data in this study were collected between February and March 2022, a time when the initial wave of the pandemic was coming to an end, but the Omicron variant was beginning to emerge across the world.

Given the cultural similarities between Saudi Arabia and Kuwait, it is perhaps understandable that there were no significant differences in the overall COHIP score between parents in Riyadh, Saudi Arabia, and those in Kuwait City, Kuwait. This is also in keeping with the fact that previous studies on dental caries and impacts of dental caries have shown a high prevalence of dental caries in both countries [6, 7, 21]. It is, however, interesting that parents in Kuwait City reported a significantly higher self-perception score than their counterparts in Riyadh. Furthermore, parents in Kuwait City reported a greater score across different domains and a higher overall score when compared to parents in Riyadh. These results must, however, be viewed keeping in mind the fact that the subjects in this study were not standardized for factors such as socioeconomic status, which have been shown to impact the effects of dental caries [22, 23].

When the impacts of age on OHRQoL were evaluated separately in Riyadh and Kuwait City, it was observed that while there was no correlation between age and OHRQoL in Riyadh there was a significant negative correlation across most domains of the COHIP in Kuwait. A similar finding was observed when the association of gender was examined separately in the two cities. It was observed that while there were no significant gender differences in Riyadh, parents of girls gave significantly higher scores across different domains in Kuwait. The reasons for this require further study and a greater standardization of the sample.

The factors for disruption of dental care during the pandemic in Kuwait and Saudi Arabia have been previously documented [14, 15]. While the study in Kuwait reported that the emphasis was on returning the level and quality of care to prepandemic levels [15], the study from Saudi Arabia noted that there was an emphasis on newer preventative measures such as silver diamine fluorides and Hall crowns [14]. This difference in approach and the potential impact of such an approach on the different OHRQoL trends reported in Saudi Arabia over Kuwait are interesting and merit further study.

The results of this study need to be viewed considering certain limitations. The current study did not account for a clinical examination of the children. Furthermore, the sample was drawn from parents of children visiting the dental clinics. Therefore, the results of the study are a reflection more of the parents' overall views rather than those related to specific oral health conditions. Despite these limitations, there were significant differences between the perception of COHIP among the two countries.

## 5. Conclusion

The COVID-19 pandemic has impacted OHRQoL in both Kuwait and Saudi Arabia. The results of this study suggest that there is a greater gender difference and age correlation of OHRQoL among the population studied in Kuwait City when compared to those in Riyadh.

## Figures and Tables

**Table 1:** Demographic distribution of the population.

Country	Gender	*N*	Mean age	Standard deviation	*t*	Sig.
Kuwait City	Male	175	6.6000	1.34762	1.605	0.288
	Female	70	6.4000	1.27859		

Riyadh	Male	291	7.1237	1.42338	3.498	<0.001^*∗*^
	Female	220	6.7000	1.26093		

^*∗*^Differences significant at *p* < 0.05.

**Table 2:** Differences in COHIP scores between the two countries.

	Country				Sig. ^*∗*^
	Kuwait City		Riyadh		
	Mean	Standard error of mean	Mean	Standard error of mean	
Oral symptoms	13.17	0.36	13.05	0.27	0.797
Functional well-being	6.62	0.29	6.25	0.21	0.308
Socioeconomic well-being	6.22	0.40	5.49	0.27	0.129
School environment	5.62	0.20	5.41	0.15	0.409
Self-image	20.24	0.22	19.51	0.18	0.010 ^*∗∗*^
Overall COHIP score	51.88	1.07	49.71	0.79	0.111

^*∗*^Calculated using the Mann–Whitney *U* test.  ^*∗∗*^Differences significant at *p* < 005.

**Table 3:** Gender differences in OHRQoL.

		Male		Female		Sig. ^*∗*^
		Mean	Standard error	Mean	Standard error	
Kuwait City	Oral symptoms	12.83	0.41	14.01	0.76	0.071
	Functional well-being	6.46	0.35	7.04	0.52	0.181
	Socioeconomic well-being	5.92	0.48	6.99	0.74	0.116
	School environment	5.45	0.25	6.06	0.36	0.091
	Self-image	19.95	0.26	20.96	0.39	0.018 ^*∗∗*^
	Overall COHIP score	50.61	1.27	55.06	1.93	0.030 ^*∗∗*^

Riyadh	Oral symptoms	13.24	0.37	12.79	0.41	0.414
	Functional well-being	6.51	0.28	5.91	0.32	0.158
	Socioeconomic well-being	5.80	0.37	5.07	0.41	0.190
	School environment	5.49	0.20	5.31	0.21	0.540
	Self-image	19.38	0.24	19.67	0.27	0.433
	Overall COHIP score	50.43	1.05	48.75	1.20	0.295

^*∗*^Calculated using the Mann–Whitney *U* test.  ^*∗∗*^Differences significant at *p* < 005.

**Table 4:** Correlation between age and the different domains of OHRQoL.

		Kuwait City	Riyadh
Overall COHIP score	Correlation coefficient ^*∗*^	−0.181 ^*∗∗*^	0.035
	Sig. (two-tailed)	0.004	0.432
	*N*	245	511

Oral symptoms	Correlation coefficient ^*∗*^	−0.072	0.049
	Sig. (two-tailed)	0.260	0.269
	*N*	245	511

Functional well-being	Correlation coefficient ^*∗*^	−0.132 ^*∗∗*^	0.007
	Sig. (two-tailed)	0.039	0.882
	*N*	245	511

Socioeconomic well-being	Correlation coefficient ^*∗*^	−0.222 ^*∗∗*^	−0.002
	Sig. (two-tailed)	<0.001	0.958
	*N*	245	511

School environment	Correlation coefficient ^*∗*^	−0.191 ^*∗∗*^	0.087
	Sig. (two-tailed)	0.003	0.051
	*N*	245	511

Self-image	Correlation coefficient ^*∗*^	−0.022	−0.036
	Sig. (two-tailed)	0.732	0.422
	*N*	245	511

^*∗*^Calculated using the Spearman's rho.  ^*∗∗*^Correlation significant at *p* < 0.05.

## Data Availability

Raw data will be made available upon reasonable request to the authors.
